# Neuroprotectin D1 Protects Against Postoperative Delirium-Like Behavior in Aged Mice

**DOI:** 10.3389/fnagi.2020.582674

**Published:** 2020-11-05

**Authors:** Ying Zhou, Jiayu Wang, Xiaofeng Li, Ke Li, Lei Chen, Zongze Zhang, Mian Peng

**Affiliations:** Department of Anesthesiology, Zhongnan Hospital of Wuhan University, Wuhan, China

**Keywords:** macrophage polarization, neuroinflammation, neuroprotectin D1, specialized proresolving lipid mediators, postoperative delirium

## Abstract

Postoperative delirium (POD) is the most common postoperative complication affecting elderly patients, yet the underlying mechanism is elusive, and effective therapies are lacking. The neuroinflammation hypothesis for the pathogenesis of POD has recently emerged. Accumulating evidence is supporting the role of specialized proresolving lipid mediators (SPMs) in regulating inflammation. Neuroprotectin D1 (NPD1), a novel docosahexaenoic acid (DHA)-derived lipid mediator, has shown potent immunoresolvent and neuroprotective effects in several disease models associated with inflammation. Here, using a mouse model of POD, we investigated the role of NPD1 in postoperative cognitive impairment by assessing systemic inflammatory changes, the permeability of the blood–brain barrier (BBB), neuroinflammation, and behavior in aged mice at different time points. We report that a single dose of NPD1 prophylaxis decreased the expression of tumor necrosis factor alpha TNF-α and interleukin (IL)-6 and upregulated the expression of IL-10 in peripheral blood, the hippocampus, and the prefrontal cortex. Additionally, NPD1 limited the leakage of the BBB by increasing the expression of tight junction (TJ)-associated proteins such as ZO-1, claudin-5, and occludin. NPD1 also abolished the activation of microglia and astrocytes in the hippocampus and prefrontal cortex, which is associated with improved general and memory function after surgery. In addition, NPD1 treatment modulated the inflammatory cytokine expression profile and improved the expression of the M2 marker CD206 in lipopolysaccharide (LPS)-stimulated macrophages, which may partly explain the beneficial effects of NPD1 on inflammation. Collectively, these findings shed light on the proresolving activities of NPD1 in the pro-inflammatory milieu both *in vivo* and *in vitro* and may bring a novel therapeutic approach for POD.

## Introduction

Postoperative delirium (POD), defined as delirium occurring mainly within 1 week after surgery, is a common neuropsychiatric complication characterized by fluctuating and concurrent disturbances of attention, cognition, psychomotor behavior, emotion, and sleep–wake rhythm (Auerbach et al., 2018). POD has been linked to higher mortality, prolonged hospitalization, and an increased risk of long-term cognitive impairment (Robinson and Eiseman, 2008; Inouye et al., 2014), and it imposes an additional medical burden on governments and society (Inouye et al., 2014; Partridge et al., 2018). The morbidity of POD ranges from 14% in general medical units to 82% in intensive care units, with an increased prevalence in elderly patients in particular (Bruce et al., 2006; Marcantonio, 2011; American Geriatrics Society Expert Panel on Postoperative Delirium in Older, 2015). With the growing aging population, the number of elderly patients who need surgery/anesthesia treatments has been increasing, as well as the prevalence of POD. However, there are no effective therapies for this complication due to the undefined underlying pathophysiology.

Recent studies highlight the importance of neuroinflammation in the development of POD (Maclullich et al., 2008; Hirsch et al., 2016; Forsberg et al., 2017). Surgical trauma activates the innate immune system, leading to the systemic release of cytokines (Hirsch et al., 2016). Humoral pro-inflammatory cytokines, such as tumor necrosis factor alpha (TNF-α) and interleukin-6 (IL-6), have been reported to be associated with the leakage of the blood–brain barrier (BBB), which leads to the entry of pro-inflammatory cytokines and monocyte-derived macrophages, resulting in the activation of glia, including microglia and astroglia (Terrando et al., 2011; Hu et al., 2018). The interaction between the peripheral and central immune systems amplifies inflammation in the brain (D’Mello et al., 2009; Perry and Teeling, 2013), and the cascade of neuroinflammation induces synaptic dysfunction and neuronal apoptosis, which ultimately impairs cognitive function (Munster et al., 2011; Plaschke et al., 2016; Skvarc et al., 2018). On this basis, treatments targeting the regulation of neuroinflammation show great potential as candidate therapies for POD.

Along with passive termination of inflammation, POD resolution actively participates in the restoration of acute inflammation as a coordinated process, which is regulated by specialized proresolving lipid mediators (SPMs; Serhan et al., 2014). SPMs are endogenously biosynthesized from essential fatty acids with potent anti-inflammatory and immunoregulatory properties (Serhan et al., 2002; Hong et al., 2003). Protectin D (PD), which is known as neuroprotectin D1 (NPD1) when synthesized in the neural system, is one of the SPMs derived from omega-3-polyunsaturated fatty acid docosahexaenoic acid (DHA). NPD1 shares biological activities with other lipid mediators such as resolvins and maresins, including accelerating nonphlogistic macrophage phagocytosis, inhibiting neutrophil infiltration, and regulating the production of cytokines and chemokines (Serhan et al., 2002; Mukherjee et al., 2004; Hong et al., 2014). Additionally, NPD1 has been demonstrated to be neuroprotective in preclinical models of Alzheimer’s disease, which shares some characteristics with POD, such as memory impairment (Lukiw et al., 2005; Safavynia and Goldstein, 2019).

Based on these discoveries, we proposed the hypothesis that prophylaxis with NPD1 could improve cognitive behavior in a POD model of laparotomy in aged mice through its proresolving effect on inflammation induced by surgical trauma. To validate this hypothesis, we assessed the natural and learned behaviors of aged mice with or without NPD1 pretreatment and the inflammation events both in the periphery and in the central nervous system (CNS). Furthermore, we aimed to determine whether NPD1 exerts anti-inflammatory and proresolving properties by promoting macrophage polarization, which is pivotal in promoting the restorative process in acute inflammation.

## Materials and Methods

### Animals

The experimental protocol was approved by the Animal Ethics Committee of Zhongnan Hospital of Wuhan University, and all experiments were performed in accordance with the National Institutes of Health Guidelines for the Care and Use of Laboratory Animals. Female C57BL/6 mice (Changsha Tianqin Biotechnology Company Limited, Changsha, China; 18 months old and weighing 30–40 g) were group-housed with four to five mice per cage under a 12-h light/dark cycle in a temperature-controlled (25 ± 2°C) room with free access to standard rodent water and food.

### Surgical Model

The mice were randomly divided into the control group, surgery group, NPD1 group, or NPD1+surgery group. NPD1 (Cayman Chemical, Ann Arbor, MI, USA) at 2 μg/ml in saline with 1.4% ethanol was administered i.p. at a dose of 600 ng (300 μl) per mouse in the NPD1 group and NPD1+surgery group, while an equal volume of 1.4% ethanol in saline was administered in the control group and surgery group. The NPD1 dose was based on studies using other models of acute inflammation with slight modification (Li et al., 2014; Yang et al., 2019). One hour after administration of NPD1 or vehicle, the mice in the surgery group and NPD1+surgery group were subjected to a simple laparotomy under isoflurane anesthesia. Specifically, each mouse was induced with 1.4% isoflurane in 100% oxygen in a transparent acrylic chamber. Fifteen minutes after induction, the mouse was removed from the chamber and placed on a heating pad to maintain body temperature between 36 and 37°C during the surgery. Isoflurane anesthesia was maintained *via* a cone device with a 16-gauge needle sensor monitoring the concentration of isoflurane. A longitudinal midline incision was made from the xiphoid to 0.5 cm proximal to the pubic symphysis through the skin, abdominal muscles, and peritoneum. Abdominal organs were partially exposed for 2 min, and the incision was then sutured layer by layer with 5-0 Vicryl thread. The procedure for each mouse lasted approximately 10 min, and the mouse was then returned to the anesthesia chamber for up to 2 h to receive the rest of the anesthesia. Blood pressure was monitored with a mouse-tail blood pressure cuff (Softron BP-2010A, Softron Beijing Biotechnology Company Limited Beijing, China), and blood gas and blood glucose levels were tested with a blood gas analyzer (i-STAT, Abbott Point of Care Inc., Princeton, NJ, USA). Analgesia with EMLA cream (2.5% lidocaine and 2.5% prilocaine) was administered before skin incision, at the end of the procedure, and every 8 h for 1 day postoperatively. The mice in the control group and the NPD1 group were placed in their home cages with 100% oxygen for 2 h without surgery treatment.

### Behavioral Tests

POD is characterized by acute concurrent disturbances at different cognitive levels, including effects on natural and learned behaviors (Auerbach et al., 2018). Therefore, we performed multiple behavioral tests in the order of buried food test, open field test, and Y maze test at 24 h before the surgery (baseline) and at 6, 9, or 24 h after the surgery in groups of three mice and finished them within 50 min, to mimic the certain features of clinical diagnosis of POD in patients, which were described in our previous studies (Peng et al., 2016).

To evaluate the natural tendency of mice to use olfactory cues, buried food tests were performed. Two days before the test, each mouse was given one to two pieces of sweetened cereal. The mice were placed within their home cage in the testing room for at least 1 h prior to testing to allow them to habituate to the environment. During habituation, the test cage was prepared by filling it with 3-cm-deep clean bedding, and a piece of sweetened cereal was randomly buried 0.5 cm below the surface of the bedding. Then, the mouse was placed in the center of the test cage for 5 min, and the latency to eat the food was measured as the time required for the mouse to uncover the food pellet and grasp it in its forepaws and/or teeth. If the mouse failed to find the pellet within 5 min, the latency was defined as 300 s.

Then, mice underwent testing in the open field test to measure their exploratory and general activity. Each mouse was placed in the center of an open field chamber (40 × 40 × 40 cm) in a quiet, illuminated room and allowed to freely explore the chamber for 5 min. The movement parameters of the mouse were monitored and analyzed *via* a video camera connected to the Any-Maze animal tracking system software (Xinruan Information Technology Company Limited, Shanghai, China). Parameters of the total distance moved, freezing time, and time spent in the center were recorded and analyzed.

To further assess spatial learning and memory ability following surgery/anesthesia, the Y maze test was also executed in a two-trial task. The Y maze apparatus consisted of three arms (width 8 × length 30 × height 15 cm) positioned at 120° angles extending from a central space, and each wall of the arms was pasted with cardboard in different patterns as visual cues. The three arms of the Y maze were randomly allocated as the novel arm, which was blocked in the first trial but opened in the second trial; the start arm, in which the mouse started to explore; and the other arm was always open. The first trial was the training trial, which allowed the mouse to explore the start arm and the other arm for 10 min, with the novel arm being blocked. After 2 h (for the tests 6 and 24 h after surgery) or 4 h (for the tests 9 h after surgery), the second trial was conducted as the retention trial. The mouse was again placed in the maze in the same start arm with free access to all three arms for 5 min. A video camera linked to the Any-Maze animal tracking system software was installed 60 cm above the chamber to monitor and analyze the number of entries and the time spent in each arm.

### Enzyme-Linked Immunosorbent Assay

The hippocampus and prefrontal cortex are two major structures involved in cognitive impairment (Flores et al., 2016a,b). Therefore, we examined the changes in inflammatory cytokines not only at the periphery but also in the hippocampus and prefrontal cortex. The concentrations of TNF-α, IL-6, and IL-10 in the plasma and brain tissues of mice at 6, 9, and 24 h after surgery were determined using enzyme-linked immunosorbent assay (ELISA) kits (eBioscience) according to the manufacturer’s instructions. The levels of IL-6, IL-10, and IL-12 in the culture supernatants of primary bone marrow-derived macrophages were also measured by ELISA kits (eBioscience) according to the manufacturer’s instructions after treatment.

### BBB Permeability Assay

Fluorescent dextran was used to measure BBB permeability, which was based on the established dye-injection assay with slight modification (Ben-Zvi et al., 2014; Yang et al., 2017). Specifically, 6 h after surgery, each mouse was injected intravenously with 100 μl 10-kDa dextran–Texas Red lysine fixable (4 mg/ml, Invitrogen, D1863). Fifteen minutes after injection, each mouse was anesthetized and decapitated. The brains were harvested and fixed by immersion in 4% paraformaldehyde (PFA) overnight at 4°C, then cryopreserved in 30% sucrose and frozen in TissueTek OCT (Sakura). Frozen sections of 20 μm were collected and postfixed in 4% PFA at room temperature (20–25°C) for 15 min, washed in PBS, blocked with 10% goat serum (Boster Biologic Technology, China) for 2 h, permeabilized with 0.5% Triton X-100, and then incubated with isolectin B4 (20 μg/ml, I21411, Molecular Probes, San Francisco, CA, USA) for immunostaining to visualize blood vessels. A Zeiss LSM 510 META microscope was used to obtain fluorescence images of the injected tracer and isolectin under a 40× objective lens. For each mouse, 20 images of 10 different slices of the hippocampus and prefrontal cortex were randomly selected, and the level of dextran found outside the vessels was analyzed using ImageJ (NIH).

Spectrophotometric quantification of 10-kDa dextran–Texas Red from the extracts of the hippocampus and prefrontal cortex was carried out at the same time point. Specifically, each mouse was injected intravenously with 100 μl 10-kDa dextran–Texas Red lysine fixable (4 mg/ml, Invitrogen, D1863) at 6 h after surgery. Fifteen minutes after injection, each of the mice was deeply anesthetized and perfused with phosphate-buffered saline (PBS) transcardially (150 ml for 5 min). Then, the mice were decapitated, and the hippocampal and prefrontal cortex tissues were harvested and homogenized in 1% Triton X-100 in PBS (100 μl/100 mg brain tissue). Tissue lysates were centrifuged at 16,000 rpm for 20 min, and the relative fluorescence of the supernatant was measured on a POLARstar Omega fluorometer (BMG LABTECH; ex/em 595/615 nm).

### Western Blot Analysis

At 6 and 9 h after surgery, mice were anesthetized and decapitated to harvest the hippocampus and prefrontal cortex tissues. Total protein samples from the brain tissues were homogenized using RIPA lysis buffer (150 mM NaCl, 1 mM EDTA, 50 mM Tris, 1% Triton, 0.1% sodium dodecyl sulfate, and 0.5% deoxycholate) containing protease and phosphatase inhibitors. The lysate was centrifuged at 12,000 rpm for 5 min at 4°C to remove the sediment. The supernatants were collected, and the protein concentration was determined with a bicinchoninic acid (BCA) protein assay kit (Aspen, Wuhan, China). After the determination of protein contents, the proteins were separated by SDS-PAGE (8–12%) and then transferred to PVDF membranes (Aspen, Wuhan, China). After being blocked with 5% skim milk for 1 h at room temperature, the membranes were incubated overnight at 4°C with the following primary antibodies: anti-ZO-1 (1:500, Abcam, ab96587), anti-occludin (1:2,000, Abcam, ab167161), and anti-claudin-5 (1:500, Biorbyt, orb214680). Anti-β-actin (1:10,000, TDY Biotech, ab37168) was used to normalized and control for loading differences in the protein levels. Then, the membranes were washed three times with TBST (20 mM Tris–HCl, 150 mM NaCl, and 0.05% Tween-20) and incubated with horseradish peroxidase-conjugated goat anti-rabbit secondary antibody (1:10,000, ASPEN, AS1107) for 0.5 h at room temperature. Specific immunoreactivity was detected using enhanced chemiluminescence (Aspen, Wuhan, China), and the signal intensity was measured using image analysis software (AlphaEaseFC software).

### Immunofluorescence

At 24 h after surgery, mice were deeply anesthetized with isoflurane and perfused transcardially with ice-cold 0.1 M PBS followed by 4% PFA in 0.1 M PBS at pH 7.4. Their brains were harvested and postfixed in 4% PFA in 0.1 M PBS at 4°C overnight, and then cryoprotected in 0.1 M PBS containing 30% sucrose for 72 h. The brains were freeze-mounted in TissueTek OCT (Sakura) and were cut sequentially to 20-μm-thick coronal sections. After washing in PBS and permeabilization in 0.5% Triton X-100, the coronal sections were blocked with 10% goat serum in PBS for 2 h at room temperature to block nonspecific binding. Then, the following primary antibodies were used: mouse anti-glial fibrillary acidic protein (GFAP; 1:500, Abcam, I21411) and rabbit anti-Iba-1 (1:200, Abcam, ab178847) at 4°C overnight. For secondary detection, goat anti-mouse and goat anti-rabbit antibodies conjugated with Alexa Fluor dyes (405 and 488) from Invitrogen (1:500) were used. The immunolabeled sections were coverslipped with 40, 6-diamidino-2-phenylindole (DAPI; Invitrogen) and analyzed by microscopy (LSM5 Exciter; Zeiss, Jena, Germany). Five high magnification areas were chosen in three nonoverlapping fields randomly acquired in the hippocampal and prefrontal cortex subregions using a counting frame size of 0.4 mm^2^. The images were processed, and the area of the astrocytes and microglia was quantified using ImageJ software (NIH). The area of the selected cells was converted into a binary image using the dilation method, and the cell outline was measured. Total immunoreactivity was calculated as the percentage area density, defined as the number of pixels (positively stained areas) divided by the total number of pixels (sum of positively and negatively stained areas) in the imaged field.

### Primary Cell Culture and Grouping

Bone marrow-derived macrophages (BMDMs) were purchased from iCell Bioscience Inc. (MIC-iCELL-i017, Shanghai, China) and cultured in DMEM/F-12 containing 10% fetal bovine serum (FBS), 100 μg/ml streptomycin, and 100 U/ml penicillin and supplementary factor (iCell Bioscience Inc., PriMed-iCell-011) at 37°C under 5% CO_2_ and 95% air. Cells were then stimulated with 10 ng/ml lipopolysaccharide (LPS, Sigma, L2880) with or without NPD1 (80 ng/ml) for 24 h. Batches of the same BMDMs were left untreated as a control.

### Flow Cytometry Analysis

For cell staining, anti-mouse CD16/CD32-PE-Cy7 and CD206-PE (Invitrogen) were used. The cells were detached and suspended in flow cytometry staining buffer and treated with Fc-receptor blocker antibody for 20 min at 4°C to eliminate nonspecific binding. Then, the cells were stained with these antibodies for 30 min at 4°C in the dark. After washing twice in flow cytometry staining buffer and resuspension in the staining buffer, samples were analyzed using a BD FACSCalibur system.

### Statistical Analysis

The sample size reported in each of the figure legends was based on our preliminary studies and was calculated by power analysis using G*Power v 3.1 to ensure a power = 0.8 and *P* < 0.05. The statistical analyses were performed with SPSS 19.0 (IBM, New York, NY, USA) or GraphPad Prism 6 (GraphPad, New York, NY, USA). Quantitative data are expressed as the means ± standard error of the mean (SEM). Statistical significance was determined using one-way or two-way analysis of variance (ANOVA), followed by the Bonferroni *post hoc* test. A *P*-value of less than 0.05 was considered statistically significant.

## Results

### POD-Like Behavior Induced by Surgery/Anesthesia in Aged Mice Is Improved by NPD1 Prophylaxis

To determine whether surgery/anesthesia affects general and cognitive behavior of aged mice, we performed a battery of behavioral tests with the food buried test, open field test, and Y maze test at 24 h before surgery and 6, 9, and 24 h after surgery in the present study as we previously reported (Peng et al., 2016; Lu et al., 2020).

**Figure 1:**
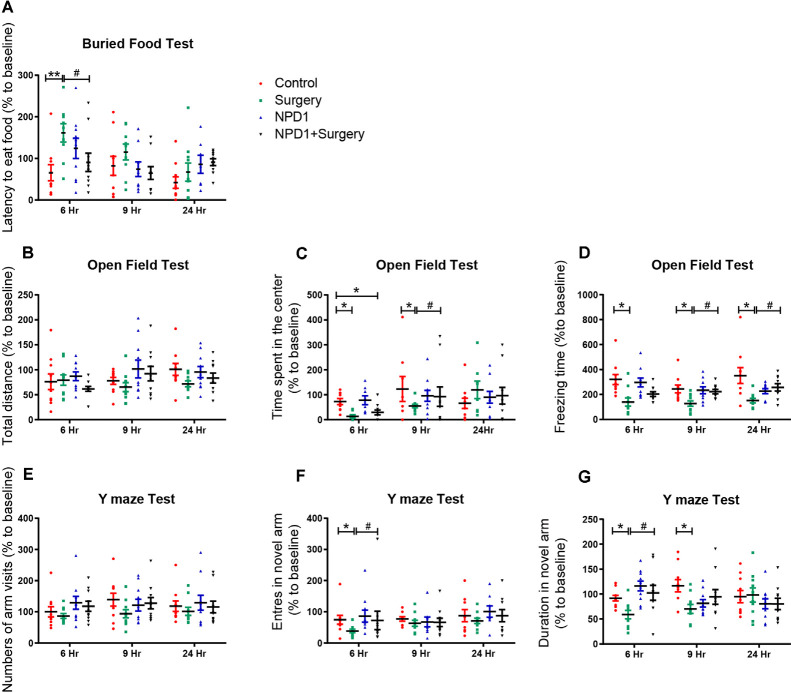
Surgery/anesthesia induces postoperative delirium (POD)-like behavior of aged mice, which can be ameliorated by preemptive administration of neuroprotectin D1 (NPD1). At 6, 9, and 24 h after surgery/anesthesia, the buried food test **(A)**, open field test **(B–D)**, and Y maze test **(E–G)** were executed ordinally. Data are presented as individuals and the lines mark the mean ± standard error of the mean (SEM). Statistics: two-way analysis of variance (ANOVA) followed by Bonferroni *post hoc* comparison. **(A–G)** *n* = 8–10 per group. *P < 0.05 vs. the control group, **P < 0.01 vs. the control group, ^#^*P* < 0.05 vs. the surgery group.

We first executed the buried food test to explore whether surgery/anesthesia affected the ability of the mice to associate an odorant with a food reward (Yang and Crawley, 2009). The latency to eat food was markedly increased in the surgery group compared with the control group at 6 h after surgery [210.3 (46.4) vs. 65.7 (19.2)%, *P* < 0.01, Figure 1A], while pretreatment with NPD1 (600 ng/mouse) improved the impaired ability to find and eat food induced by surgery/anesthesia [90.7 (22.0)% vs. surgery, *P* < 0.05, Figure 1A]. No significant changes were observed between the NPD1 group and the control group.

Then, we executed the open field test to examine the locomotor ability and exploratory behavior of the mice subjected to surgery/anesthesia or NPD1 treatment (Gould et al., 2009). There were no significant differences in the total distance traveled by the mice among the four groups at any time point (*P* > 0.05, Figure 1B), indicating that surgery/anesthesia did not affect the motor function of aged mice. Surgery/anesthesia significantly decreased the time spent in the center at 6 and 9 h after surgery [11.0 (5.3)% vs. control and 55.0 (7.3)% vs. control, *P* < 0.05, Figure 1C], and the preemptive administration of NPD1 alleviated this phenomenon at 9 h after surgery [92.5 (15.2)% vs. control, *P* < 0.05, Figure 1C]. In addition, surgery/anesthesia significantly decreased the freezing time at 6, 9, and 24 h after surgery [139.4 (33.5)% vs. control, 127.2 (21.6)% vs. control, 151.7 (18.3)% vs. control, *P* < 0.05, Figure 1D], while preoperative treatment with NPD1 increased the freezing time at 9 and 24 h after surgery [223.0 (14.1)% vs. surgery, 256.9 (29.3)% vs. surgery, *P* < 0.05, Figure 1D]. NPD1 administration alone did not change these parameters compared with the control condition (*P* > 0.05, Figures 1B–D).

Finally, we conducted the Y maze test to assess hippocampus-dependent spatial memory in aged mice as previously validated (Wheelan et al., 2015). Surgery/anesthesia did not alter the number of arm visits among the four groups (*P* > 0.05, Figure 1E). However, surgery/anesthesia significantly reduced the number of entries into the novel arm at 6 h after surgery [38.6 (4.3)% vs. control, *P* < 0.05, Figure 1F] and the duration spent in the novel arm at 6 and 9 h after surgery [58.8 (5.2)% vs. control, 69.9 (5.4)% vs. control, *P* < 0.05, Figure 1G] compared with the control condition. Pretreatment with NPD1 increased the number of entries into the novel arm and the duration spent in the novel arm at 6 h after surgery [10.2.5 (6.9)% vs. surgery, *P* < 0.05, Figures 1F,G]. NPD1 administration *per se* did not affect the performance of aged mice in the Y maze test at any time point.

In conclusion, prophylaxis with NPD1 attenuated the impairment of general behavior (buried food test and open field test) and learned behaviors (Y maze test) induced by surgery/anesthesia in aged mice in a time-dependent order.

### NPD1 Modulates the Expression of Inflammatory Cytokines After Surgery Both at the Periphery and in the CNS

**Figure 2:**
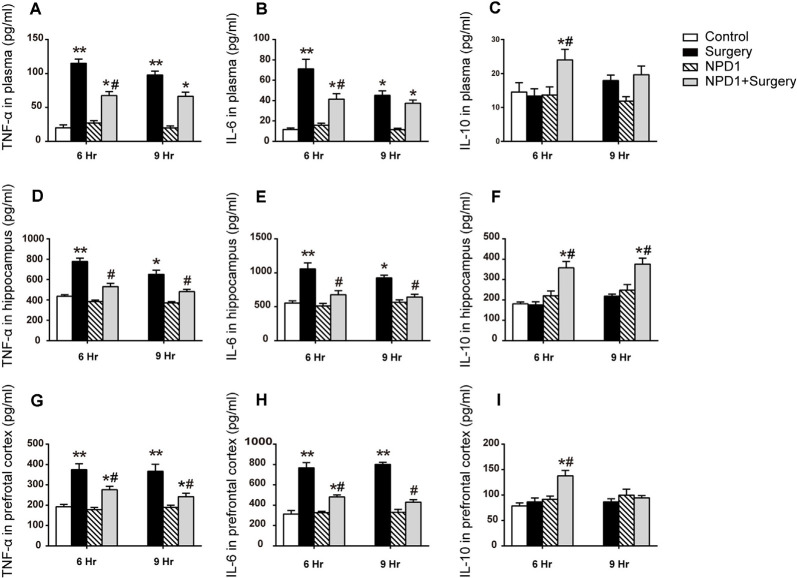
Effects of NPD1 on the expression of inflammatory cytokines *in vivo*. NPD1 pretreatment alleviated the surgery-induced upregulation of pro-inflammatory factors and promoted the expression of anti-inflammatory factors both in the peripheral blood and the central locations such as the hippocampus and prefrontal cortex at different time points **(A–I)**. The cytokines in peripheral blood and brain tissues were measured by enzyme-linked immunosorbent assay (ELISA). Data are presented as mean ± SEM. Statistics: two-way ANOVA followed by Bonferroni *post hoc* comparison. **(A–I)** *n* = 4–5 per group. *P < 0.05 vs. the control group, **P < 0.01 vs. the control group, ^#^*P* < 0.05 vs. the surgery group.

To assess the effects of NPD1 on systemic inflammation and neuroinflammation, we first measured the changes in TNF-α, IL-6, and IL-10 in blood plasma after surgery. Surgery/anesthesia significantly increased the levels of TNF-α and IL-6 at 6 and 9 h after surgery (*P* < 0.05, Figures 2A,B) but did not change the expression of IL-10 (*P* > 0.05, Figure 2C). Although a single dose of NPD1 did not completely reverse the increase in pro-inflammatory cytokines to the control condition, it markedly reduced the levels of TNF-α and IL-6 at 6 h after surgery (*P* < 0.05, Figures 2A,B). In addition, pretreatment with NPD1 increased the expression of IL-10, a crucial cytokine during the resolution phase of inflammation, at 6 h after surgery (*P* < 0.05, Figure 2C). Second, we measured these cytokines in the hippocampus and prefrontal cortex, two key brain regions related to the memory network (Place et al., 2016). Surgery/anesthesia induced a marked increase in the expression of TNF-α and IL-6 at 6 and 9 h after surgery in both the hippocampus and prefrontal cortex compared with the control condition (*P* < 0.05, Figures 2D,E,G,H). Prophylaxis NPD1 significantly decreased the expression of TNF-α and IL-6 at 6 and 9 h compared with the surgery group in these brain regions (*P* < 0.05, Figures 2D,E,G,H). Notably, pretreatment with NPD1 increased the expression of IL-10 not only in the hippocampus at 6 and 9 h after surgery (*P* < 0.05, Figure 2F) but also in the prefrontal cortex at 6 h after surgery (*P* < 0.05, Figure 2I). No effects on these cytokines were reported when treated with NPD1 alone.

### NPD1 Prophylaxis Alleviated the Leakage of the BBB Induced by Surgery/Anesthesia in Aged Mice

The breakdown of BBB has been reported to be associated with delirium and perioperative neurocognitive disorders (Maldonado, 2008; Subramaniyan and Terrando, 2019). Herein, we employed a well-established dye injection assay to investigate the integrity of the BBB (Ben-Zvi et al., 2014; Yang et al., 2017) under treatment of surgery/anesthesia with or without NPD1.

**Figure 3:**
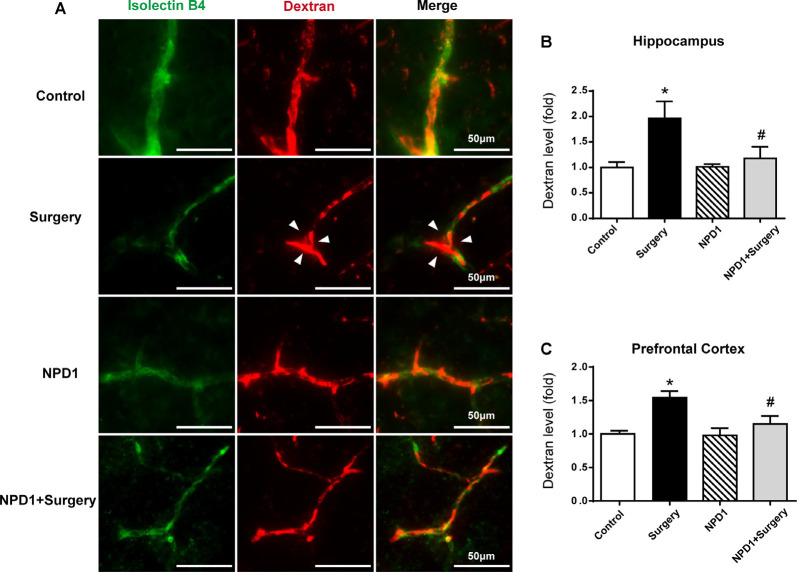
NPD1 protects against the leakage of the blood–brain barrier (BBB) induced by surgery/anesthesia in the hippocampus and prefrontal cortex. Immunostaining of blood vessels (isolectin B4, green) and intravenously injected dextran (10 kDa, red) in brain sections of the hippocampus at 6 h after surgery **(A)**. The arrowhead marked area indicates that the dextran was extravascular. The spectrophotometric quantification of extravascular dextran (10 kDa) levels in the extraction of the hippocampus and prefrontal cortex showed that surgery/anesthesia increased the permeability of the BBB compared with the control, and pretreatment with NPD1 attenuated this phenomenon **(B,C)**. Data are presented as mean ± SEM. Statistics: two-way ANOVA followed by Bonferroni *post hoc* comparison. **(B,C**) *n* = 4–5 per group. *P < 0.05 vs. the control group, ^#^*P* < 0.05 vs. the surgery group. Scale bars represent 50 μm in **(A)**.

Immunofluorescence images revealed that 10-kDa dextran was primarily confined to vessels in the control group, NPD1 group, and NPD1+surgery group. By contrast, the dextran signal was detected in the brain parenchyma around vessels in the surgery group (Figure 3A). To quantify the extravascular dextran, spectrophotometric quantification of 10-kDa dextran–Texas Red from brain tissue extracts was performed. In both the hippocampus and prefrontal cortex, we found that surgery/anesthesia increased the level of extravascular 10-kDa dextran compared with the control condition, while NPD1 prophylaxis decreased the leakage of dextran induced by surgery/anesthesia (*P* < 0.05, Figures 3B,C).

**Figure 4:**
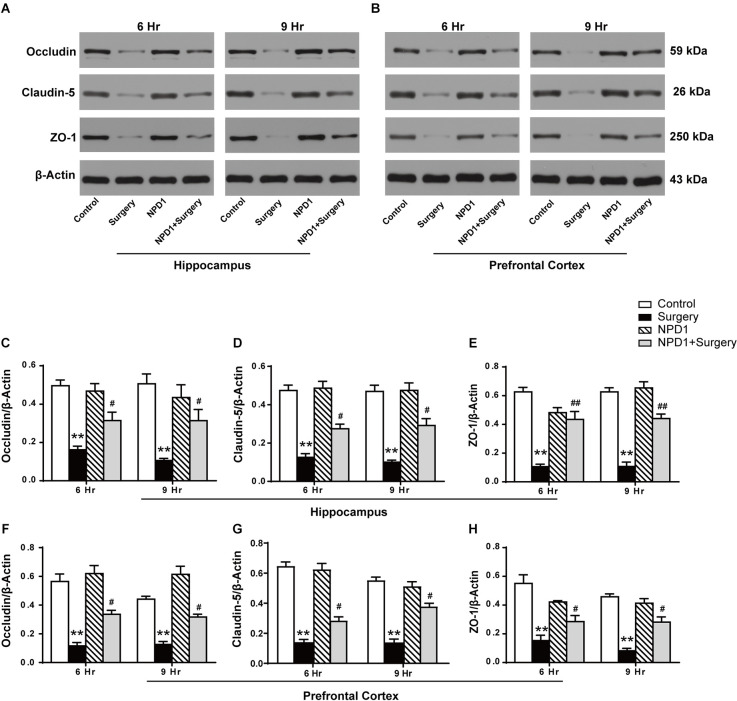
NPD1 modulates the expression of tight junction (TJ)-associated proteins in the hippocampus and prefrontal cortex after surgery. Representative Western blotting bands of the expression of occludin, claudin-5, and ZO-1 in the hippocampus and prefrontal cortex at 6 and 9 h after surgery **(A,B)**. Quantification analyses of the expression of occludin, claudin-5, and ZO-1 were normalized to that of β-actin as internal control **(C–H)**. Data are presented as mean ± SEM. Statistics: two-way ANOVA followed by Bonferroni *post hoc* comparison. **(C–H)** *n* = 4–5 per group. **P < 0.01 vs. the control group, ^#^*P* < 0.05 vs. the surgery group, ^##^*P* < 0.01 vs. the surgery group.

We next examined the effects of NPD1 on the expression of occludin, claudin-5, and ZO-1 after surgery, which are tight junction (TJ)-associated proteins that maintain the integrity of the BBB (Jiao et al., 2011, Figures 4A,B). By quantitative Western blotting, we found that there was a marked decrease in the expression of ZO-1, claudin-5, and occludin in both the hippocampus and prefrontal cortex at 6 and 9 h after surgery, while pretreatment with NPD1 significantly attenuated the reduction of these proteins (*P* < 0.05, Figures 4C–H). Preemptive administration of NPD1 alone did not change the homeostasis of the BBB.

### NPD1 Reverses the Reactive States of Astrocytes and Microglia in the Hippocampus and Prefrontal Cortex

**Figure 5:**
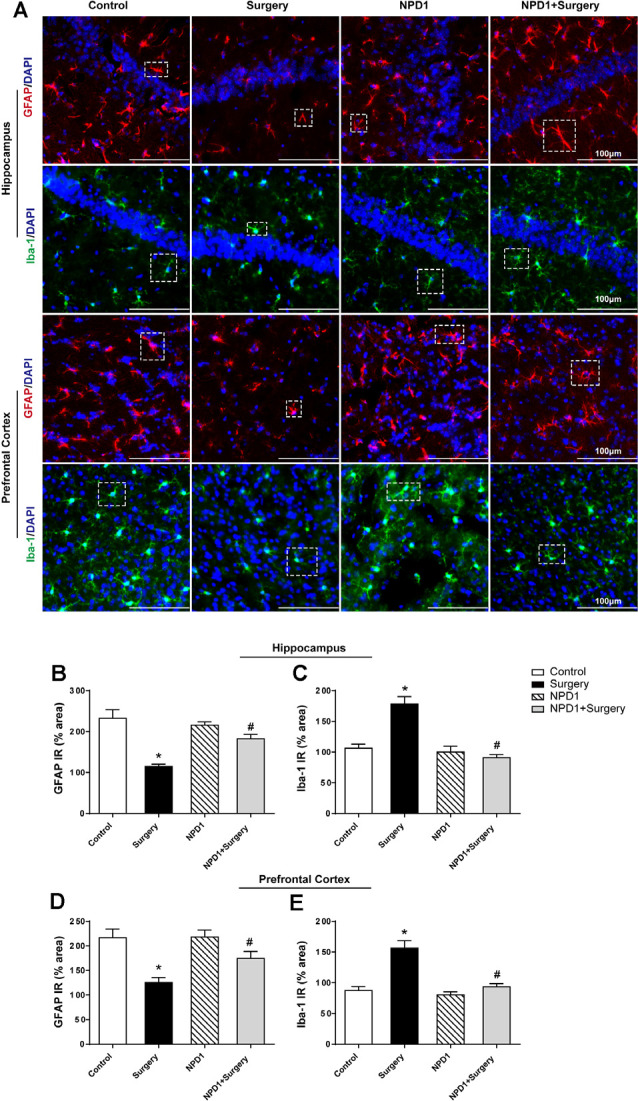
NPD1 pretreatment reverses the activation of astrocytes and microglia in the hippocampus and prefrontal cortex elicited by surgery/anesthesia. Representative images of immunofluorescence showed the expression of glial fibrillary acidic protein (GFAP) and Iba-1 in the hippocampus and prefrontal cortex at 24 h after surgery **(A)**. Surgery/anesthesia caused distinct changes in the morphology of glial cell, including shortened processes and a reduced cell soma in astrocytes and an amoeba-like morphology in microglia. Preemptive administration of NPD1 significantly restored the classic stellate shape of astrocytes and the ramified shape of microglia. Quantification results of immunostaining at 24 h after surgery shown in **(B–E)**. Data are presented as mean ± SEM. Statistics: two-way ANOVA followed by Bonferroni *post hoc* comparison. **(B–E)** *n* = 4–5 per group. *P < 0.05 vs. the control group, ^#^*P* < 0.05 vs. the surgery group. Scale bars represent 100 μm in **(A)**.

We measured the changes in the immunoreactivity of GFAP and Iba-1 in the hippocampus and prefrontal cortex to assess the reactive states of microglia and astrocytes, which represent the major pathological manifestation of neuroinflammation (Terrando et al., 2013; Norden et al., 2016; Joshi et al., 2019). Astrocytes in the hippocampus and prefrontal cortex showed significant morphological changes, including shorter and deramified processes, an atrophic cell soma, and a reduced GFAP immunoreactive area after surgery compared with the control condition (*P* < 0.05, Figures 5A,B,D). By contrast, the mice that underwent surgery but were pretreated with NPD1 retained the stellate shape of classical astrocytes, with longer processes and similar immunoreactive areas to those of the control group (*P* < 0.05, Figures 5A,B,D).

NPD1 also attenuated microglial activation, as measured by changes in the expression of Iba-1. Surgery induced an amoeba-like morphology of microglia and increased the Iba-1 immunoreactive area in the hippocampus and prefrontal cortex compared with the control condition (*P* < 0.05, Figures 5A,C,E), while preemptive administration of NPD1 significantly restored the ramified shape of microglia and reduced the cellular area (*P* < 0.05, Figures 5A,C,E). There were no significant changes in GFAP or Iba-1 in the NPD1 group.

### NPD1 Alleviates the Production of Pro-inflammatory Cytokines and Promotes the Macrophage Polarization Toward M2 in the LPS-Stimulated BMDMs

**Figure 6:**
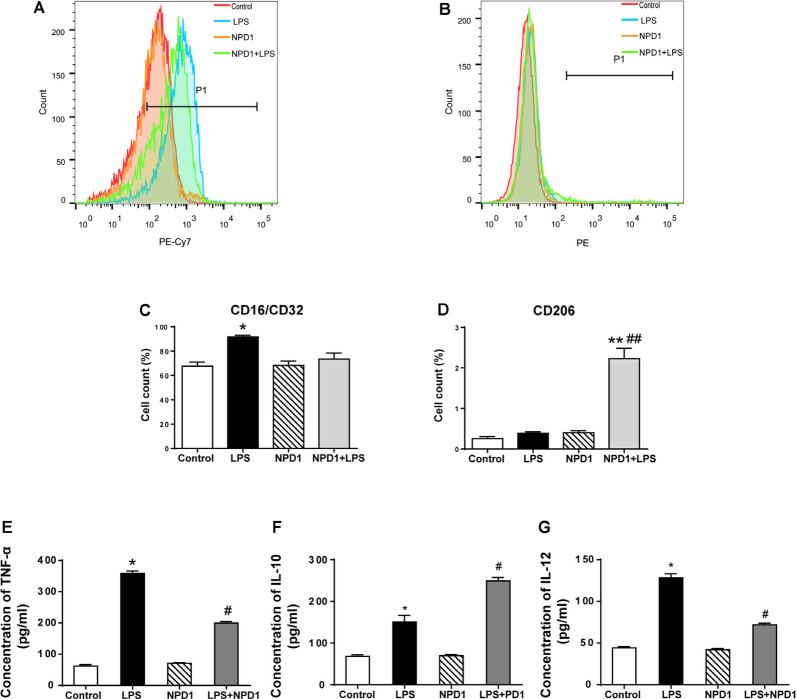
NPD1 promotes macrophage polarization to the M2 phenotype after pro-inflammatory stimulation of BMDMs and modulates the expression of inflammatory cytokines. NPD1 promoted the transformation of BMDM cell markers from the M1 to M2 phenotypes, as detected by flow cytometry **(A,B)**. The ratios of M1 and M2 phenotypes in different groups were calculated **(C,D)**. The production of tumor necrosis factor-α (TNF-α), interleukin (IL)-12, and IL-10 by BMDMs were tested by ELISA **(E–G)**. Data are presented as mean ± SEM. Statistics: two-way ANOVA followed by Bonferroni *post hoc* comparison. **(A–G)** *n* = 5 per group. *P < 0.05 vs. the control group, **P < 0.01 vs. the control group, ^#^*P* < 0.05 vs. the surgery group, ^##^*P* < 0.01 vs. the surgery group.

NPD1 has been reported to exert a proresolving effect *via* immunoregulation, including blocking neutrophil infiltration and promoting phagocytosis *in vivo* (Hong et al., 2003; Ariel and Serhan, 2007), which is related to the reaction of polarized macrophages (Mosser and Edwards, 2008; Tabas, 2010). To better investigate the effects of NPD1 on macrophage polarization, we cultured LPS-stimulated BMDMs with or without NPD1. The polarization of BMDMs was analyzed on the basis of the expression of the M1 marker CD16/CD32 and the M2 marker CD206 (Figures 6A,B). Quantitative flow cytometry analysis showed that the M1 population was significantly increased in the LPS group compared with the control group (*P* < 0.05, Figure 6C). NPD1 coincubation tended to downregulate macrophage polarization to M1 and markedly increased the M2 population compared with LPS incubation alone (*P* < 0.01, Figures 6C,D). The levels of TNF-α and IL-12 were significantly increased by LPS stimulation (*P* < 0.05, Figures 6E,G), while coincubation with NPD1 decreased the production of these two cytokines by LPS-stimulated BMDMs (*P* < 0.05, Figures 6E,G). Incubation with LPS alone increased the level of IL-10 compared with the control group (*P* < 0.05, Figure 6F), suggesting the spontaneous initiation of inflammation resolution, but coculture with NPD1 increased the expression of IL-10 (*P* < 0.05, Figure 6F).

## Discussion

In the present study, we demonstrate that NPD1, a novel lipid-derived mediator of SPMs, contributes to the postoperative recovery of POD-like behavior in aged mice through its anti-inflammatory and proresolving effects. Our results indicate that prophylaxis with NPD1 at peripheral injury sites alleviates the systemic inflammatory response and protects BBB integrity after laparotomy. Moreover, it limits neuroinflammation in both the hippocampus and prefrontal cortex, according to the expression of inflammatory cytokines and the reactive states of microglia and astroglia in these brain regions. These protective actions against inflammation displayed by NPD1 may be related to macrophage polarization toward M2, as we showed in the *in vitro* experiment. To the best of our knowledge, this is the first report of the effects of NPD1 in a rodent model of POD.

Cumulative evidence has revealed the pivotal role of neuroinflammation in the occurrence of POD, while peripheral inflammation is considered to represent the initiation of neuroinflammation (Terrando et al., 2010; Groh and Martini, 2017; Subramaniyan and Terrando, 2019). In the aseptic surgery setting, injured cells activate BMDMs by releasing damage-associated molecular patterns (DAMPs) that bind to Toll-like receptors (TLRs) of BMDMs, thereby upregulating the expression of pro-inflammatory cytokines such as TNF-α, IL-1β and IL-6 (Akira and Takeda, 2004; Lotze and Tracey, 2005; Zhang et al., 2010). These cytokines can cause further activation of DAMPs through positive feedback, ultimately leading to increased inflammation (Gabay et al., 2010; Terrando et al., 2010). Our results demonstrated that NPD1 attenuated the systemic postsurgery release of TNF-α and IL-6, which are the pivotal cytokines that appear after trauma (Terrando et al., 2010). These findings are consistent with the potent anti-inflammatory activity of NPD1 in many other disease models that are associated with inflammation, such as peritonitis (Ariel et al., 2006), corneal damage (Lukiw et al., 2005), asthma (Levy et al., 2007), and inflammatory pain (Bang et al., 2018). In a murine peritonitis model, PD1/NPD1 has been proven to effectively attenuate polymorphonuclear neutrophil infiltration and the expression of pro-inflammatory cytokines even at a very small dose (1 ng/mouse; Hong et al., 2003; Ariel et al., 2005, 2006). Furthermore, the preemptive administration of NPD1 increases the systemic expression of IL-10, which is one of the most important mediators of inflammatory resolution as a potent suppressor of classical macrophage activation (Mosser and Zhang, 2008). It has been demonstrated that inflammatory cytokines, such as TNF-α, IL-6, IL-1β, and IL-12, are strictly associated with M1-like macrophages, while IL-10 is primarily secreted by M2-like macrophages (Mosser and Edwards, 2008; Murray, 2016). The changes in the cytokine profile in the periphery suggest that macrophage polarization toward M2 may be linked with NPD1.

The breakdown of the BBB is seen as a hallmark of neuroinflammation because its disruption facilitates the infiltration of peripheral immunocompetent cells and cytokines into the immunologically privileged brain (Galea et al., 2007; He et al., 2012). The barrier function of the BBB is mainly attributed to the TJs of brain microvascular endothelial cells (Pardridge, 2005). TNF-α and IL-6 have been reported to disturb the integrity of the BBB by reducing the expression of TJ-associated proteins between the neurovascular endothelium (Rochfort et al., 2015; Blecharz-Lang et al., 2018). Notably, TNF-α can upregulate cyclooxygenase 2 isozyme (COX2) in the brain microvascular endothelium, thereby increasing the local generation of prostaglandins, which exhibit a potent ability to increase vascular permeability (Engblom et al., 2002; Rajakariar et al., 2006). NPD1 and its precursor DHA show protective activity related to the BBB and neurocognitive behavior after experimental ischemic stroke (Belayev et al., 2011, 2018), which was also noted in our model. The restoration of the impaired BBB by preemptive NPD1 administration may be indirect, resulting from the modulated profile of cytokines in the circulation that we discussed above, similar to the mechanism whereby NPD1 alleviates leakage under laser-induced choroidal neovascularization (Sheets et al., 2010). Interestingly, the synthesis of PDs is an enzymatic process that occurs *via* a mechanism involving lipoxygenase (LOX), and the transcription of LOX is initiated by the same signaling pathways involved in producing prostaglandins E2 and D2 (Bannenberg et al., 2005; Rajakariar et al., 2006). The production of IL-10 also requires the participation of prostaglandins (Mosser and Zhang, 2008). This kind of temporal–spatial interaction between inflammatory mediators can at least partly explain why there is no valid evidence that inflammatory inhibitors can be used to treat POD or other neurocognitive disorders because they may hinder the resolution phase of inflammation. Therefore, NPD1, as well as other SPMs, may be desirable therapies for inflammation-driven diseases.

In addition to mitigating the peripheral inflammatory response, NPD1 reduced the activation of glial cells and the expression of inflammatory cytokines in the hippocampus and prefrontal cortex. As resident macrophages in the CNS, microglia play a role in immune surveillance and respond to different kinds of pathological stimuli (Kettenmann et al., 2013). Once activated, microglia rapidly switch to a pro-inflammatory phenotype with a stout morphology and enhance the production of pro-inflammatory molecules such as IL-1α, TNF-α and complement component 1q (C1q; Clausen et al., 2008; Liddelow et al., 2017). These specific cytokines, along with cell debris released by classically activated microglia, can trigger the transformation of astroglia to A1, the detrimental reactive phenotype of astrocytes (Norden et al., 2016; Liddelow et al., 2017; Joshi et al., 2019). A1 astrocytes lose their supportive abilities in the CNS (i.e., maintaining synaptic functions and phagocytic capacity) and simultaneously secrete neurotoxins to induce neuronal death (Gómez-Galán et al., 2012; Liddelow et al., 2017). In our model of POD, NPD1 reversed the morphological changes in microglia and astrocytes in both the hippocampus and prefrontal cortex to their original states, representing restorative transformation from the inflamed phenotype to the resting states, and thus modified the pro-inflammatory milieu by modulating the secretion of inflammatory cytokines. It is thus not surprising that NPD1 pretreatment facilitates the recovery of POD-like behavior in aged mice because these two profitable brain regions act in concert to shape emotion, learning, and memory organization and transform information (Eichenbaum, 2017; Tyng et al., 2017). Although microglia share similar properties with peripheral macrophages, they may not be the target affected by NPD1. Recently, parkin-associated endothelin-like receptor (Pael-R), also known as GPR37, has been identified as the specific receptor for NPD1 (Bang et al., 2018). GPR37 is enriched in oligodendrocytes and astrocytes but not microglia (Cahoy et al., 2008; Bang et al., 2018). In this context, the anti-inflammatory and proresolving effects of NPD1 in the CNS may be mediated by different cell types, and the underlying mechanism requires further investigation.

BMDMs have been shown to be the bridge that links the peripheral and central immune systems since they can infiltrate the brain in conditions characterized by neuroinflammation (Tanaka et al., 2003; Liu et al., 2008). Their function can be deleterious or favorable, depending on their polarization states in relation to the extracellular milieu (Italiani and Boraschi, 2014; Murray, 2016). Other members of SPMs derived from the same precursor as NPD1 have been shown to induce M2 polarization (Titos et al., 2011; Marcon et al., 2013; Akagi et al., 2015), which suggests that a similar property may exist in NPD1. Actually, we demonstrated the phenotypic skewing of inflammatory mediators promoted by NPD1 *in vitro* toward attenuating the M1 macrophage markers (TNF-α and IL-12) and elevating the M2 macrophage marker (IL-10). The shift in specific cell receptors on LPS-stimulated BMDMs also verified that M2 polarization was induced by NPD1. These findings suggest that the proresolving effect of NPD1 is linked to the transformation of macrophage polarization toward the M2 phenotype. However, three subsets of the M2 phenotype, designated M2a, M2b, and M2c, each of which has different protective properties, have been identified within the M2 phenotype (Biswas and Mantovani, 2010). It is thus essential to further explore the specific effect of NPD1 on macrophages and inflammation.

There are several limitations to our research. First, we emphasized the role of NPD1 in humoral mechanisms for active inflammation resolution in the present study. The vagus nerve also participates in this process by controlling the expression of netrin-1, which exerts a synergistic effect with SPMs (Mirakaj et al., 2014). The integration of multiple signaling pathways of NPD1 requires further investigation in the perioperative context. Second, we only used local analgesics to control incisional pain, which may not be effective in attenuating nociceptive stimuli. Additionally, NPD1 has been reported to reverse inflammatory pain induced by the i.p. injection of zymosan (Bang et al., 2018). In the absence of any pain-related behavioral tests in the present study, it was difficult to determine whether NPD1 enhanced the postoperative recovery of mice by acting in an additive manner with local analgesics to relieve pain in our study. Assessments of pain behaviors will be executed in our future research. Third, we only detected the biochemical events of neuroinflammation, including cellular constituents in the CNS and morphological changes in glial cells, but not the influence of NPD1 intervention on neurons. NPD1 can provide protection to improve the survival of neural cells (Calandria et al., 2015; Belayev et al., 2017). In addition, glia–neuron cross talk, especially in the hippocampus, is highly involved in the normal function of neurons to form memory and consciousness (Chung et al., 2015; Santello et al., 2019). For this reason, the different modes of action in different cell types need to be further illuminated.

In conclusion, the present study identifies the novel role of NPD1 in relieving the POD-like behavior of aged mice and regulating postoperative inflammation not only in the periphery but also in the hippocampus and prefrontal cortex. These protective effects of NPD1 may be related to its modulation of macrophage polarization, which needs further investigation. Collectively, these findings indicate the potential of NPD1 to be a novel therapy for neuroinflammation and POD.

## Data Availability Statement

The raw data supporting the conclusions of this article will be made available by the authors, without undue reservation.

## Ethics Statement

The animal study was reviewed and approved by Animal Ethics Committee of Zhongnan Hospital of Wuhan University.

## Author Contributions

YZ and JW designed and performed the experiment, collected and analyzed the data, and prepared the manuscript. XL and JW was involved in preparing the animal models and participated in interpreting the results. KL contributed to behavioral testing. LC was involved in biochemical analysis. YZ and JW participated in the statistical analysis. MP contributed to the study concept and design, secured funding for the project, and prepared and critically revised the manuscript. All authors contributed to the article and approved the submitted version.

## Conflict of Interest

The authors declare that the research was conducted in the absence of any commercial or financial relationships that could be construed as a potential conflict of interest.
