# Corrigendum: Neuroprotectin D1 protects against postoperative delirium-like behavior in aged mice

**DOI:** 10.3389/fnagi.2022.934855

**Published:** 2022-09-05

**Authors:** Ying Zhou, Jiayu Wang, Xiaofeng Li, Ke Li, Lei Chen, Zongze Zhang, Mian Peng

**Affiliations:** Department of Anesthesiology, Zhongnan Hospital of Wuhan University, Wuhan, China

**Keywords:** macrophage polarization, neuroinflammation, neuroprotectin D1, specialized proresolving lipid mediators, postoperative delirium

In the original article, there was a mistake in Figure 4 as published. The bar graph for Claudin-5 protein expression (Figure 4G) incorrectly repeated the bar graph for Occludin expression (Figure 4F). The corrected Figure 4 appears below.

**Figure 4:**
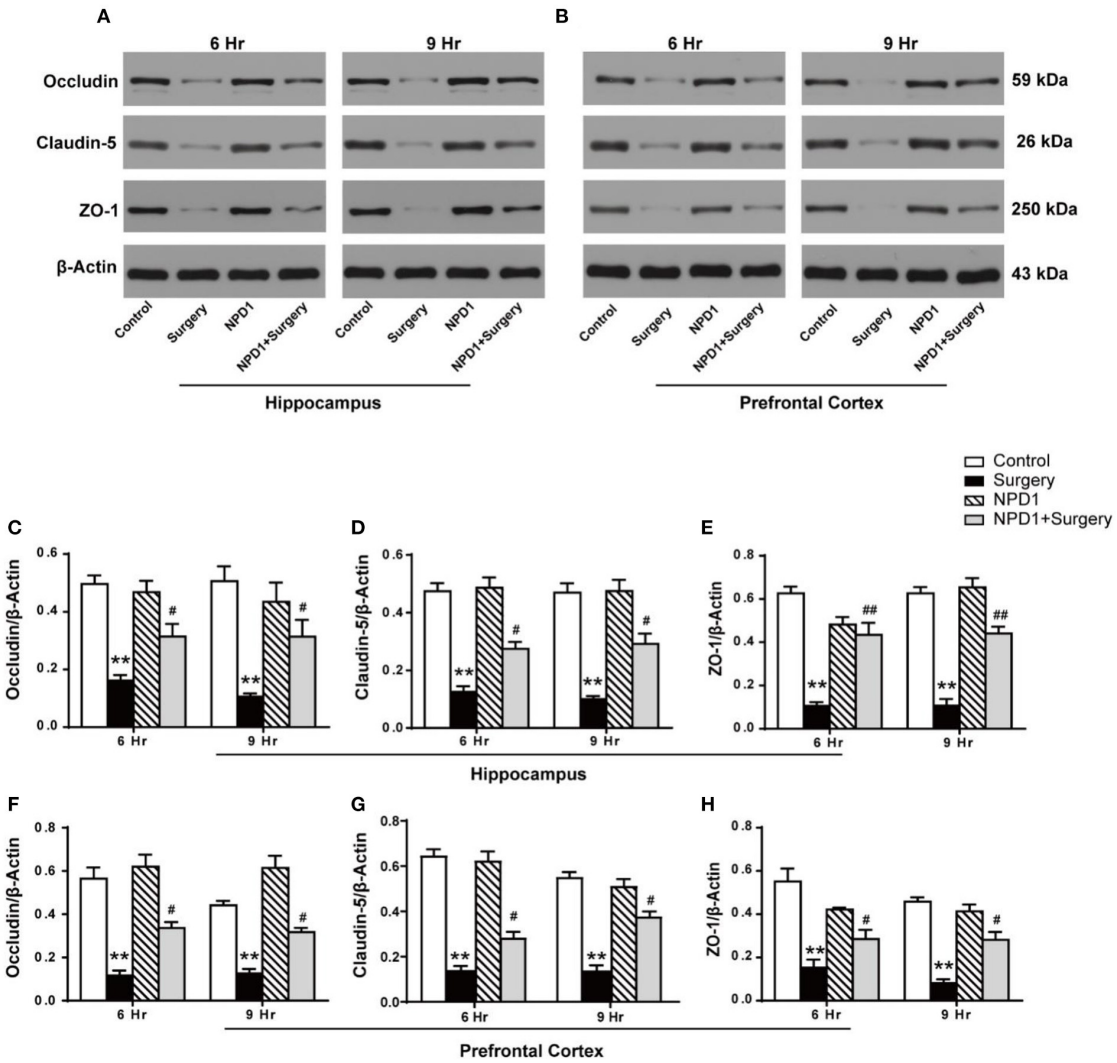
NPD1 modulates the expression of tight junction (TJ)-associated proteins in the hippocampus and prefrontal cortex after surgery. Representative Western blotting bands of the expression of occludin, claudin-5, and ZO-1 in the hippocampus and prefrontal cortex at 6 and 9 h after surgery **(A,B)**. Quantification analyses of the expression of occludin, claudin-5, and ZO-1 were normalized to that of β-actin as internal control **(C–H)**. Data are presented as mean ± SEM. Statistics: two-way ANOVA followed by Bonferroni *post hoc* comparison. **(C–H)** *n* = 4–5 per group. ^**^P < 0.01 vs. the control group, ^#^*P* < 0.05 vs. the surgery group, ^##^*P* < 0.01 vs. the surgery group.

The authors apologize for this error and state that this does not change the scientific conclusions of the article in any way. The original article has been updated.

## Publisher's note

All claims expressed in this article are solely those of the authors and do not necessarily represent those of their affiliated organizations, or those of the publisher, the editors and the reviewers. Any product that may be evaluated in this article, or claim that may be made by its manufacturer, is not guaranteed or endorsed by the publisher.

